# Haplotyping pharmacogenes using TLA combined with Illumina or Nanopore sequencing

**DOI:** 10.1038/s41598-022-22499-0

**Published:** 2022-10-22

**Authors:** Laurentijn Tilleman, Kaat Rubben, Wim Van Criekinge, Dieter Deforce, Filip Van Nieuwerburgh

**Affiliations:** 1 Laboratory of Pharmaceutical Biotechnology, Ghent University, Ottergemsesteenweg 460, 9000 Ghent, Belgium; 2 Laboratory of Bioinformatics and Computational Genomics, Ghent University, Coupure Links 653, 9000 Ghent, Belgium

**Keywords:** Genetics, Genotype, Haplotypes, Sequencing

## Abstract

The currently used pharmacogenetic genotyping assays offer limited haplotype information, which can potentially cause specific functional effects to be missed. This study tested if Targeted Locus Amplification (TLA), when using non-patient-specific primers combined with Illumina or Nanopore sequencing, can offer an advantage in terms of accurate phasing. The TLA method selectively amplifies and sequences entire genes based on crosslinking DNA in close physical proximity. This way, DNA fragments that were initially further apart in the genome are ligated into one molecule, making it possible to sequence distant variants within one short read. In this study, four pharmacogenes, *CYP2D6*, *CYP2C19*, *CYP1A2* and *BRCA1*, were sequenced after enrichment using different primer pairs. Only 24% or 38% of the nucleotides mapped on target when using Illumina or Nanopore sequencing, respectively. With an average depth of more than 1000X for the regions of interest, none of the genes were entirely covered with either sequencing method. For three of the four genes, less than half of the variants were phased correctly compared to the reference. The Nanopore dataset with the optimized primer pair for *CYP2D6* resulted in the correct haplotype, showing that this method can be used for reliable genotyping and phasing of pharmacogenes but does require patient-specific primer design and optimization to be effective.

## Introduction

Genotyping is one of the most important aspects of personalized medicine, particularly within the pharmacogenetic field^1,2^. In many medical disciplines, pharmacogenetic genotyping is used to predict a patient’s phenotype to adjust therapy^3,4^. Especially the genetic variation in drug-metabolizing enzymes significantly contributes to the differing benefit-risk balance of certain drugs between patients^1,4^. As the importance of personalized medicine became increasingly apparent during the last decades, numerous assays have been developed to genotype pharmacogenes accurately.

However, most pharmacogenetic testing platforms are currently limited to a small number of targeted variants with known functional effects^5^. The effects of these assayed variants are primarily interpreted individually, without knowing if other variants occur on the same allele. Although some functional consequences can be predicted from these individual variants, the gene’s overall function is determined by the combination of all variants per allele, including those in introns and promotor regions. Therefore, complete haplotypes are often critical for translating genetic results into accurate, medically actionable findings^6^. As PGx genotyping is usually done using targeted short-read massively parallel sequencing (SR-MPS) technologies, resolving haplotype information of the generated data is not evident^7^. It typically requires supplemental statistical phasing based on known haplotype structure in the population, which is error-prone, or on parental genotypic data, which is not always available^8^.

De Vree et al.^9^ developed a new targeting method called Targeted Locus Amplification (TLA) that could be instrumental in overcoming this phasing problem. This method selectively amplifies and sequences entire genes based on crosslinking DNA in close physical proximity. It enables the generation of DNA fragments containing sequences that were initially further apart in the genome, making it possible to sequence distant variants within one short read. In different input cells, distinct combined fragments of distant sequences are generated. Moreover, as homologous chromosomes are physically separated in eukaryotic cells, TLA provides DNA fragments originating from only one of the homologous chromosomes. Therefore, sequencing these TLA libraries allows relatively straightforward phasing of distant variants when primer sets are designed considering their position relative to allele discriminating SNVs in the studied samples^9–12^. However, performing a personalized primer design for each patient is not desirable for a pharmacogenetic assay to be used in practice. This study aims to test the use of TLA for many samples without prior knowledge of SNV positions by using generic TLA primers in combination with the TLA kit. In previously reported studies, TLA libraries were mainly sequenced using Illumina sequencing. However, Illumina sequencing can only generate reads with one, two, or sporadically a few more joined sequences. In contrast to this short-read sequencing technology, long-read sequencing technologies, such as Nanopore sequencing, could generate reads with more than ten of these concatenated sequences, thereby increasing the efficiency of phasing more distant variants without increasing the number of viewpoints. To study whether Illumina or Nanopore performs best for variant calling and phasing of TLA libraries when using a limited number of generic primers not specifically close to allele-specific SNVs, the TLA libraries of the four pharmacogenes described below were sequenced with both sequencing technologies, and the datasets were compared afterward.

This study performed TLA sequencing on four important pharmacogenes: *CYP2D6, CYP2C19, CYP1A2* and *BRCA1*. The cytochrome P450 (CYP) genes are important pharmacogenes and affect about 75% of the current drugs^13^. *CYP2D6* is one of the most widely studied pharmacogenes because of its contribution to the Phase 1 metabolism of about 25% of all clinically prescribed drugs and its high genetic variability within and between populations^4^. Therefore, accurate genotyping assays for this gene are of major importance. Although *CYP2D6* is a relatively small gene spanning only 4400 nucleotides, accurate genotyping of this gene is challenging. First of all, the *CYP2D6* gene is surrounded by two pseudogenes showing 94% sequence similarity with *CYP2D6*, which complicates the genotyping of this gene. Furthermore, *CYP2D6* is one of the most polymorphic human genes, with over 130 star(*)-alleles and over 400 sub-alleles defined by the Pharmacogene Variation (PharmVar) Consortium^14,15^. Most *CYP2D6* star alleles are defined by specific combinations of single nucleotides variants (SNVs) and small insertions and deletions (INDELs). In addition, the *CYP2D6* gene locus often contains complex structural variants, such as complete gene deletions, gene duplications or multiplications, or hybrid tandem rearrangements with the highly similar *CYP2D7*. Due to this complexity, it is challenging to analyze *CYP2D6* by conventional short-read sequencing methods without linking information. Another widely studied pharmacogene is *CYP2C19*, which contributes to the metabolism of many clinically relevant drug classes. This gene is approximately a hundred thousand base pairs long and has more than 30 described pharmacogenetic variants^16^. Moreover, more than 60 clinical pharmacogenetic associations with several drugs have been described. *CYP1A2* is a smaller gene of about 10,000 base pairs and was included to study the performance of TLA on a shorter gene when using only one viewpoint. Lastly, the *BRCA1* gene was included in this study as successful TLA libraries were already generated in previous studies^9^.

## Material and methods

### Viewpoint design

To test the use of generic TLA primers for haplotyping multiple samples without prior knowledge of SNV positions, the TLA Primer Design Manual was followed, describing the design rules for generic TLA primers. The primers were designed in unique regions of the genes and were located within a fragment between two NlaIII restriction cut sites. As *CYP2D6*, *CYP2D7* and the sequences upstream and downstream of these genes are highly similar to each other, not many positions are available to design viewpoints for that pharmacogene. Designing viewpoints near known common SNVs in these few unique regions is challenging and emphasizes the advantage of using generic primer pairs, independent of known common SNVs, for complicated genes such as *CYP2D6*. Different primer pair positions, or viewpoints, were designed for *CYP2D6*, *CYP2C19* and *CYP1A2* by using the NCBI Primer-BLAST tool. For *CYP2D6*, four primer pairs were designed. Because of the high homology with *CYP2D7* and *CYP2D8*, these primer pairs were first tested, and the best primer pair without enrichment for *CYP2D7* and *CYP2D8* was chosen for the experiments. For *CYP2C19*, four primer pairs were designed as well. For this gene, we designed primer pairs evenly spread throughout the gene to maximize the coverage over the length of the gene. For *CYP1A2,* only one primer pair was designed. For *BRCA1*, one primer pair that resulted in good coverage over the gene was provided by Cergentis. However, this primer pair is not located near known allele-specific SNVs. Therefore, our results for *BRCA1* cannot be compared directly with the previously generated phasing results in de Vree et al*.*^9^, which uses other primer pairs for the phasing purpose. The primer sequences (IDT, Coralville, IA, USA) are provided in Table S1.

### TLA workflow

**Figure 1:**
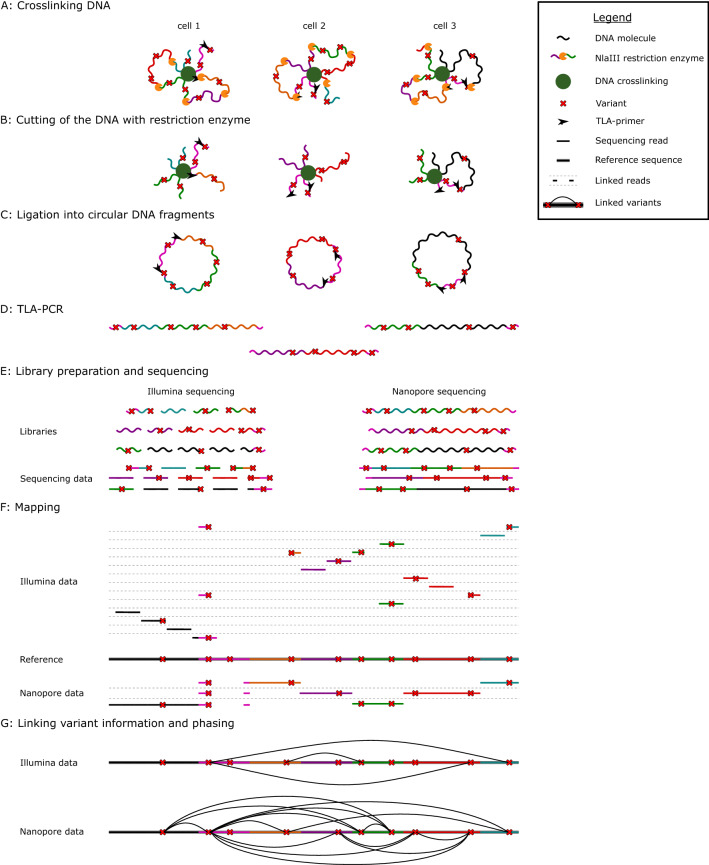
Overview of the TLA workflow performed in this study. (**A**): First, the DNA of each cell was crosslinked; (**B**): Then, the DNA was digested with the NlaIII restriction enzyme; (**C**): Next, the restricted DNA fragments were ligated into circular DNA, and circular DNA strands were reverse crosslinked; (**D**): A TLA-PCR was performed using the designed primers, resulting in linear DNA; (**E**): The DNA was split into two pools, one for Illumina sequencing and one for Nanopore sequencing. On each pool, corresponding library preparations and sequencing were performed. (**F**): Illumina and Nanopore sequencing data were mapped on the GRCh38 reference genome; (**G**): After variant calling, the link information in the sequencing data was used to link the different variants enabling a more detailed phasing.

The TLA cell kit (Cergentis, Utrecht, The Netherlands) was executed following the TLA Protocol for cell lines, which is identical to what de Vree et al.^9^ conducted in their study, as shown in Fig. 1. The TLA workflow was performed on the GM12878 cell line (Coriell Institute for Medical Research, Camden, NY, USA), which was cultured according to the standard protocol. 4.8 × 10^6^ fresh cultured cells were crosslinked using formaldehyde. Then the DNA was digested with the NlaIII restriction enzyme, followed by ligation and reversed crosslinking. Next, the DNA molecules were digested with NspI and ligated at a low DNA concentration (5 ng/µL). This way, intramolecular ligation was promoted to obtain circular chimeric DNA molecules for PCR amplification. To check if every step was completed correctly, the DNA length was measured on a 1% agarose E-Gel EX With SYBR Gold II (Thermo Fisher, Waltham, MA, USA) before digesting, after digesting, and after ligation (Fig. S1). Separate TLA PCRs were performed with each primer pair on 600 ng of the TLA template. The quality of the PCR products was measured afterward using a High Sensitivity DNA chip (Agilent Technologies, Santa Clara, CA, US) (Fig. S2).

The different PCR products from each viewpoint were sequenced on an Illumina and a Nanopore sequencer. The Illumina libraries were prepared using the Nextera DNA Flex Library Prep Kit (Illumina, San Diego, CA, USA), with 300 ng PCR product as input material, using five PCR cycles to introduce the barcodes. Libraries were pooled as defined in Table S2 and were sequenced on two Illumina MiSeq Micro paired-end 150 bp runs. The Nanopore libraries were prepared according to the Nanopore Protocol ‘Amplicons by Ligation (SQK-LSK109)’. The PCR products of the different viewpoints were pooled in three different pools as defined in Table S2. 500 ng and 1 µg of the pools were used as input for the Flongle and the MinION library preparation, respectively. The first two pools were sequenced on an R9.4.1 Flongle Flowcell by loading 20 fmol of the final library. The last pool was sequenced on an R9.4.1 MinIon Flowcell using 40 fmol of the final library. All data was basecalled using the GUPPY super accuracy algorithm version 6.0.1.

### Data analysis

The Illumina libraries were demultiplexed based on the barcodes introduced during the library preparation, giving rise to ten separate libraries, one for each viewpoint. Reads were then trimmed with cutadapt version 1.15, using a quality score above 20 and a minimum read length of 15 bp^17^. Trimmed reads were mapped as single-end sequenced reads with BWA SW with a mismatch penalty of seven to the human reference genome GRCh38^18^. Reads that did not map correctly were split at the NlaIII recognition sequences closest to the clipping-end that was still mapped. The parts of the split reads that were not mapped were mapped again with BWA SW with a mismatch penalty of seven. Reads that were not appropriately mapped after the second mapping round were further split and mapped until all reads were mapped or could not be further split. Variants were called with GATK version 4.1.4.0. Only variants detected with a depth of 25 were further used. Filtered variants were phased using the updated phasing algorithm of Cergentis ^12,19^.

Nanopore sequencing data from the different runs were analyzed together, without demultiplexing the different viewpoints. Reads were first mapped with minimap2 to the human reference genome GRCh38^20^. Reads that did not map correctly were split at the NlaIII recognition sequences closest to the clipping-end that was still mapped. The parts of the split reads that were not mapped were mapped again with minimap2. Reads that were not appropriately mapped after the second mapping round were further split and mapped until all reads were mapped or could not be further split. Variants were called using clair3^21^. Only variants detected with a depth of 100 were retained. Filtered variants were phased with Whatshap version 1.2.1^22^. Phased blocks were further phased using an adapted phasing algorithm of Cergentis^12,19^.

Phased variants of the different genes were benchmarked against the phased reference of the GM12878 cell line described by Krusche et al*.*^23^ using hap.py and custom Python scripts^19,24^.

The SNV frequency was not quantified in this study as we used a cell line to assess the performance of TLA for reliable genotyping and phasing. However, in many applications like cancer biopsies, it is important to know at what percentage a certain SNV occurs in a tissue. The capability of TLA to quantify the SNV frequency is well described in de Vree et al*.*^9^. They could quantify SNV percentages ranging from 5 to 95%.

## Results

### General sequencing results

As the different genes are located on different chromosomes, it can be assumed that enriched reads for one gene will not interfere with the data analysis of another gene. Nevertheless, Illumina data were demultiplexed per viewpoint as barcodes were introduced during the library preparation. The demultiplexed Illumina data for the different viewpoints of one gene were combined and analyzed together. The data for the Nanopore runs were not demultiplexed. The number of reads generated by both techniques can be found in Table 1. Although Nanopore sequencing resulted in fewer sequenced reads than Illumina sequencing, both techniques generated about the same number of sequenced nucleotides.

**Table 1:** Number of sequenced reads and nucleotides from the Illumina and Nanopore sequencing runs.

Sequencing device	Gene	Sequenced reads	Sequenced nucleotides
Illumina	*CYP2D6*	6,081,688	852,872,370
	*CYP2C19*	4,471,944	627,365,650
	*CYP1A2*	1,860,141	266,789,864
	*BRCA1*	2,690,711	393,168,924
	All genes	15,104,484	2,140,196,808
Nanopore	All genes	1,339,697	2,076,058,625

Table 2 shows that Nanopore sequencing generated a higher percentage of on-target mapped nucleotides than Illumina sequencing. This higher on-target mapping percentage can be attributed to the longer sequencing length of the Nanopore reads. The longer the read length, the better a read can be mapped to the correct position. The Illumina data were demultiplexed for each viewpoint, resulting in separate on-target percentages for each gene. For the long genes, *CYP2C19* and *BRCA1,* 15% and 41% of the nucleotides mapped on-target, whereas for the shorter genes *CYP1A2* and *CYP2D6,* 31% and 15% of the nucleotides mapped on-target, respectively.

**Table 2:** Number and percentage of nucleotides on-target and average depths.

	Illumina	Depth Illumina	Nanopore	Depth Nanopore
*CYP2D6*	126,139,274	29,260 X	105,719,746	24,523 X
*CYP2C19*	256,904,994	2795 X	544,052,362	5919 X
*CYP1A2*	81,396,219	10,487 X	14,690,029	1893 X
*BRCA1*	57,832,947	7167 X	114,459,735	14,185 X
Percentage on-target	24.40%		37.52%	

For reliable variant calling, minimum required depths of 25 and 100 reads were set for Illumina and Nanopore sequencing, respectively. Although the average depth on all genes largely exceeds these minimum depths, only *CYP2D6* was covered entirely at these depths when combining the data from all the viewpoints (Table 3). These coverage differences can be attributed to the TLA technology, as it amplifies the DNA using primers that bind on the viewpoints. Consequently, the DNA fragments containing the viewpoint are always amplified, but other parts of the DNA are only amplified when they are ligated to these fragments. The sequencing depth is thus expected to be highest around the viewpoints and drop rapidly when moving further away from the viewpoints. The regions where not a single read was mapped were mainly located between two NlaIII recognition sites close to each other, generating small fragments that pose difficulties during the mapping process. These fragments could only be mapped if one of the surrounding recognition sites was not cut and the flanking region was covered. Therefore, increasing the sequencing depth will not result in better coverage of these regions. As variants cannot be called or phased in these regions, this constitutes one of the major drawbacks of the TLA technology.

**Table 3:** Number of positions covered at the minimum depth, 25X for Illumina and 100X for Nanopore.

	Gene length	Covered Illumina at 25X (%)	Covered Nanopore at 100X (%)
*CYP2D6* (all view points)	4311	4311 (100.0)	4311 (100.0)
*CYP2D6* (viewpoint 4)	4311	4292 (99.6)	4300 (99.7)
*CYP2C19*	91,918	86,252 (93.8)	84,945 (92.4)
*CYP1A2*	7762	7740 (99.7)	2627 (33.8)
*BRCA1*	81,069	79,574 (98.2)	77,682 (95.8)

**Figure 2:**
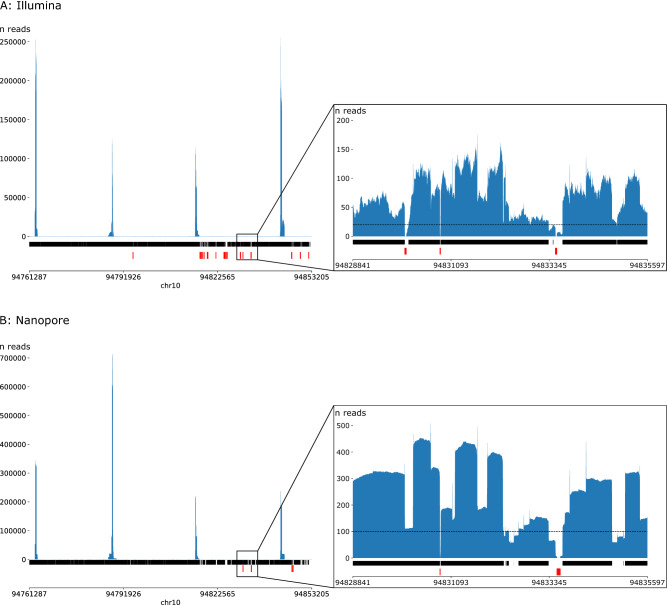
Coverage plots for *CYP2C19*. (**A**) Illumina sequencing data; (**B**): Nanopore sequencing data. Blue surfaces represent the depth on each position in *CYP2C19*; dark bars under the graphs define the regions with a minimal depth, 25 reads for Illumina sequencing and 100 reads for Nanopore sequencing; red bars under the graphs represent regions with no reads.

However, obtaining the best possible coverage with TLA can be done by designing multiple viewpoints spread evenly across the gene of interest. This was done to increase the coverage of *CYP2C19,* the largest gene in this study, as we designed four viewpoints for that gene. Figure 2 shows that the depth over *CYP2C19* is not equally distributed and increases nearby the viewpoints as expected from TLA data. However, the coverage plots show that by using multiple viewpoints, almost all positions of the gene could be covered. Adding more viewpoints could possibly further increase the number of positions covered with the minimum required depth. However, fragments between two NlaIII recognition sites closer to each other than the space needed to design the TLA primers still remain an issue, as they rely on viewpoints designed in neighboring fragments. Therefore, there is no guarantee that these fragments will be covered when more viewpoints are used.

For *CYP2D6,* we also used multiple viewpoints, as designing specific primers for this gene is difficult due to the high homology between *CYP2D6* and *CYP2D7*. The viewpoints were first tested to determine if they were unique for *CYP2D6*. As shown in Fig. S3, only viewpoint 4 was specific for *CYP2D6*. The other viewpoints also show enrichment of *CYP2D7* and *CYP2D8*. However, as *CYP2D6* was not entirely covered when only data from viewpoint 4 were used, both the data from viewpoint 4 and the combined data from all the viewpoints are discussed for *CYP2D6* (Table 3).

The coverage for the Illumina dataset of *CYP1A2* is in line with the results of the other sequenced genes. However, for the Nanopore dataset of *CYP1A2*, only 33.8% of the gene is covered. The lower number of nucleotides sequenced for this dataset causes this low percentage of covered positions for the Nanopore dataset of *CYP1A2* (Table 2). Although all the TLA-PCR libraries are pooled on equal weights, ten times fewer nucleotides were sequenced for the *CYP1A2* library. This can be explained by the library length, which is slightly longer for *CYP1A2* than the other libraries (Fig. S2). Nanopore sequencing preferentially sequences shorter reads. Therefore, the libraries with shorter fragments are enriched during Nanopore sequencing, resulting in less sequenced reads of the *CYP1A2* library. As almost all nucleotides are already covered by at least one read (Fig. S4), increasing the percentage of positions covered by the minimum depth could be resolved by sequencing this sample separately or adjusting the pooling ratio considering the amplicon size differences to obtain a more even NGS output.

### Variant calling and phasing

In general, more positions, and therefore more variants, were covered in the Illumina datasets versus the Nanopore datasets (Table 3). The INDELs of the four genes and all the SNVs of *CYP1A2* and *CYP2D6* were covered in the Illumina datasets. In contrast, only the Nanopore dataset for *CYP2D6* covered all the variants in the reference. However, as previously stated, sequencing at a higher depth would not directly result in the coverage of more variants due to the inherent difficulty of sequencing certain positions using this TLA technology.

### *CYP2D6*

**Figure 3:**
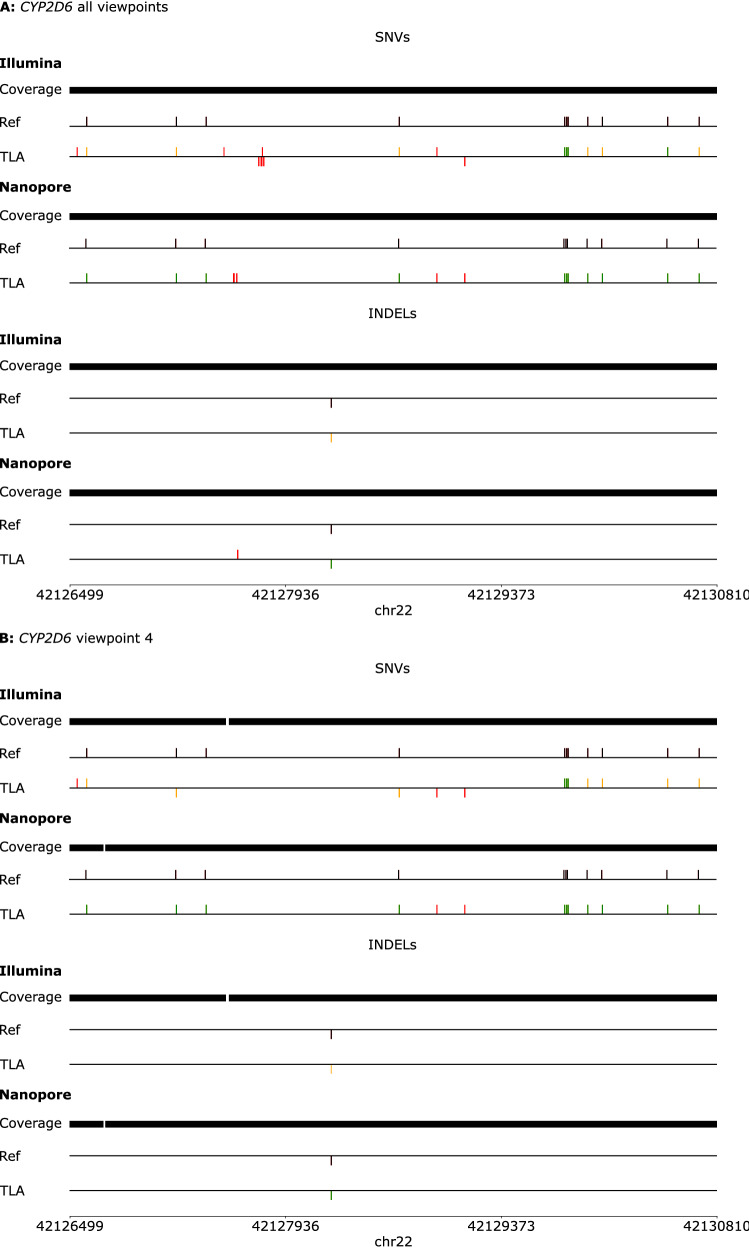
Coverage, variant calling, and phasing results for *CYP2D6*. (**A**): Results for all viewpoints of *CYP2D6*; (**B**): Results for viewpoint 4 of *CYP2D6*. Coverage: bars indicate where sufficient depth is reached: 25X for Illumina and 100X for Nanopore. Ref: The reference line shows the variants present in the reference set of Krusche et al*.*^23^. Bars plotted at the same side of the reference line (top or bottom) represent variants from the same allele. TLA: red bars represent discordant variants, orange bars represent variants that were phased incorrectly or that could not be phased, and green bars represent concordantly called and phased variants.

*CYP2D6* is one of the most challenging pharmacogenes to genotype due to the high similarity with its neighboring pseudogenes and the frequent occurrence of *CYP2D6-CYP2D7* hybrids. As shown in Fig. S3, viewpoints 1, 2 and 3 did not exclusively enrich *CYP2D6* but also *CYP2D7*, resulting in reads that mapped on both genes. Our data analysis did not remove ambiguously mapped reads, which resulted in detecting more discordant variants in the dataset from all the viewpoints (Fig. 3). In the dataset of viewpoint 4, specifically amplifying *CYP2D6*, this mapping translated into the detection of less discordant variants. In the Nanopore dataset, there were two discordant SNVs compared to the reference set of Krusche et al*.*^23^. Nevertheless, according to Rubben et al.^25^, these two SNVs are present in the GM12878 cell line but were not included in the reference set of Krusche et al*.* due to the limitations of the used sequencing techniques^23^. The Illumina dataset from viewpoint 4 also contained these SNVs in addition to one other discordant and one missing SNV. Overall, one more concordant variant was called in the Nanopore datasets than in the Illumina datasets for *CYP2D6* (Table 4, Fig. 3).

**Table 4:** Overview of the variants in the reference of Krusche et al.^23^ and the called variants in the Illumina and Nanopore datasets for each studied gene. The upper part shows the number of variants called in the Illumina datasets, while the lower part shows the number of variants in the Nanopore datasets. Covered SNVs and INDELs are the variants where the sequencing depth was at least 25X and 100X for Illumina and Nanopore datasets, respectively.

Illumina	SNVs in reference	Covered SNVs	Called SNVs	Concordant SNVs	INDELs in reference	Covered INDELs	Called INDELs	Concordant INDELs
*CYP2D6* (all viewpoints)	11	11	19	10	1	1	1	1
*CYP2D6* (viewpoint 4)	11	11	13	10	1	1	1	1
*CYP2C19*	105	99	96	94	12	11	14	9
*CYP1A2*	3	3	3	3	2	2	7	0
*BRCA1*	120	117	118	110	32	32	78	30
**Nanopore**								
*CYP2D6* (all viewpoints)	11	11	16	11	1	1	2	1
*CYP2D6* (viewpoint 4)	11	11	13	11	1	1	1	1
*CYP2C19*	105	97	79	43	12	11	12	0
*CYP1A2*	3	1	1	1	2	0	3	0
*BRCA1*	120	115	132	77	32	31	52	7

As for phasing, all the heterozygous variants in *CYP2D6* could be phased correctly in both the combined and viewpoint 4 Nanopore datasets. In contrast, only four and three of the 11 SNVs could be phased correctly in the Illumina datasets of all viewpoints and viewpoint 4, respectively, and the INDEL could not be phased in either of the datasets (Table 5). These results show the difficulty of correctly phasing the *CYP2D6* gene using Illumina sequencing for TLA libraries. The phasing difficulty can partly be explained by the fact that *CYP2D6* and *CYP2D7* are located next to each other and are highly homologous to each other. Therefore, many fragments in the TLA library contain both parts of *CYP2D6* and *CYP2D7*. Incorrect mapping of these fragments on the other gene is the leading cause of incorrect calling and phasing of the variants in *CYP2D6*. Furthermore, the differing read length between Nanopore and Illumina libraries constitutes a possible explanation for the better phasing of the former. Longer reads spanning multiple NIaIII recognition sites can be generated using Nanopore sequencing. This results in reads spanning gene-specific regions making these reads map uniquely, and the variants called and phased correctly. Our results reflect this, as *CYP2D6* could be haplotyped correctly in the Nanopore datasets. Consequently, as expected, long reads demonstrated an advantage for phasing purposes.

**Table 5:** Overview of the heterozygous variants in the reference set of Krusche et al.^23^ and the phased variants in the Illumina and Nanopore datasets for the studied genes. Concordantly called variants are shown between brackets. The upper part shows the number of variants phased in the Illumina datasets, and the lower part shows the number of variants phased in the Nanopore datasets.

Illumina	Heterozygous SNVs	Phased SNVs	Concordant phased SNVs	Heterozygous INDELs	Phased INDELs	Concordant phased INDELs
*CYP2D6* (all viewpoints)	11	4	4 (/10)	1	0	0 (/1)
*CYP2D6* (viewpoint 4)	11	5	3 (/10)	1	0	0 (/1)
*CYP2C19*	89	29	26 (/81)	11	0	0 (/7)
*BRCA1*	119	3	3 (/110)	31	0	0 (/28)
**Nanopore**						
*CYP2D6* (all viewpoints)	11	11	11 (/11)	1	1	1 (/1)
*CYP2D6* (viewpoint 4)	11	11	11 (/11)	1	1	1 (/1)
*CYP2C19*	89	29	15 (/29)	11	0	0 (/0)
*BRCA1*	119	49	36 (/77)	31	5	3 (/7)

### *CYP2C19*, *CYP1A2* and *BRCA1*

In contrast with *CYP2D6*, for *CYP2C19*, *CYP1A2* and *BRCA1,* more concordant variants were called in the Illumina datasets than in the Nanopore datasets (Table 4, Fig. S5, Fig. S6, Fig. S7). Overall, 5 to 7 of the covered variants were not called in the Illumina datasets, as opposed to almost half of the covered variants that were not called in the Nanopore datasets. Furthermore, more discordant variants were called in the Nanopore datasets compared to the Illumina datasets (Table 4).

No phasing information can be given for *CYP1A2* as this gene contains only one heterozygous variant in the reference set of Krusche et al*.*^23^. *CYP2C19* and *BRCA1* contain 89 and 119 heterozygous variants, respectively, but although almost all SNVs were called correctly in the Illumina datasets, only 30 and 3% of the heterozygous SNVs could be phased correctly (Table 5). As expected, it was impossible to phase distant SNVs for *BRCA1* in the Illumina dataset using this primer set as no SNVs were present near the viewpoint for *BRCA1,* causing an insufficient number of links between distant SNVs, resulting in only three variants that could be phased. This finding underlines the need to implement specific primer design rules when TLA is used for phasing and NGS is performed using a short-read sequencing device. In contrast, for *CYP2C19,* multiple SNVs were present on the DNA fragments of the four viewpoints. Consequently, it was possible to phase distant SNVs, as shown in Fig. S5. Overall, 90% of the phased variants in *CYP2C19* and *BRCA1* were correctly phased, referring to Krusche et al*.*^23^. None of the INDELs were phased in the Illumina datasets.

In the Nanopore datasets of *CYP2C19* and *BRCA1*, 30 and 40% of the SNVs could be phased. However, phasing seemed as good as random, as only half and 73% of the phased SNVs were phased correctly for *CYP2C19* and *BRCA1*, respectively (Table 5). As for INDELs, none of them could be phased for *CYP2C19,* and only three out of the five phased INDELs were phased correctly for *BRCA1*.

## Discussion

This study compared the performance of Illumina and Nanopore sequencing for TLA libraries of four different pharmacogenes when generic TLA primers were used. For *CYP1A2*, *CYP2C19,* and *BRCA1*, Illumina sequencing resulted in better variant calling due to the lower error rate of this sequencing technology. However, the short Illumina sequencing reads were difficult to correctly map on genes containing homologous regions, such as *CYP2D6,* which is highly homologous to its pseudogene *CYP2D7*. This was reflected in both the *CYP2D6* combined dataset of all the viewpoints and the *CYP2D6* dataset of viewpoint 4. Although the use of primers unique to the gene of interest translated into an increase of unambiguously mapped reads, as Vermeulen et al*.*^11^ also reported, incorrect variants were still called due to the remaining ambiguously mapped reads. Hence, TLA combined with Illumina sequencing did not perform sufficiently for regions of interest containing homologous regions. In contrast, long-read sequencing technologies, such as Nanopore sequencing, could resolve the problem of unambiguously mapped short reads in homologous regions. Moreover, the long reads generated by Nanopore sequencing also resulted in the correct phasing of the variants for *CYP2D6* as all variants were correctly called and phased in the Nanopore dataset of viewpoint 4.

However, due to the higher error rate coupled with the Nanopore sequencing technology, the Nanopore datasets did not consistently result in better variant calling of the other three tested genes. As shown in the *CYP2C19*, *CYP1A2*, and *BRCA1* Nanopore datasets*,* many variants were missing or called incorrectly. Nevertheless, ONT is continuously working on optimizing its sequencing accuracy, which holds the promise of obtaining better results in this context in the future. Their latest optimization led to the production of the updated SQK-LSK114 library preparation kit and R10.4.1 sequencing pore, which proved to achieve higher sequencing accuracies of over Q20^26^. Unfortunately, this updated library preparation kit and flow cells containing the updated pore were not available at the time of writing. Alternatively, an additional step could be introduced in the TLA workflow to obtain better accuracies when using Nanopore sequencing. By additionally performing rolling circle amplification on the generated TLA-PCR amplicons, the amplicons are circularized and amplified by a bacteriophage phi29 strand-displacing DNA polymerase, resulting in long reads containing multiple copies of the same PCR amplicon^27,28^. These repeats can be used to obtain a higher accuracy consensus of the original amplicon when sequenced on a Nanopore sequencing device. However, this introduces an additional step in the protocol, increasing the cost of this assay. Another possible approach is to use the SMRT long-read sequencing technology, which creates circular DNA that is read several times by the sequencing device. This platform can sequence up to 100 kb. As most of the TLA-PCR amplicons are between 4 and 10 kb, these amplicons could be read ten times using SMRT sequencing, resulting in consensus reads with accuracies above Q40^29^.

For genes without homologous regions, the better sequencing quality of Illumina resulted in better variant calling than the longer reads generated by Nanopore sequencing. However, despite the high sequencing quality and the higher number of correctly called variants in the Illumina datasets, only a minority of the called variants could be phased correctly. The design and number of viewpoints used can possibly explain this. If a viewpoint is chosen near the position of a heterozygous variant, this variant can be used to correctly phase other variants which are included after linking DNA fragments in close physical proximity^9,11,12^. As no heterozygous variant was present in the fragment of the viewpoint for *BRCA1*, this was the main cause for the low percentage of correctly phased variants for this gene. In contrast, when two out of the four viewpoints of *CYP2C19* contained a heterozygous variant in their fragments, this resulted in more phased variants for this gene. Subsequently, adding more viewpoints located in fragments containing a heterozygous variant would be beneficial to increase the number of variants that can be phased. In that regard, Vermeulen et al.^11^ first sequenced a list of 80 possible heterozygous variants for each sample, for each of which a unique viewpoint was selected. After that, only the viewpoints in fragments containing defined heterozygous variants in the sample were used. This workflow could possibly also be applied in our study to improve our results further. However, this is very time-consuming and would increase the cost of the assay. Moreover, perfect phasing would still not be guaranteed, as some heterozygous variants are located in homologous regions.

The TLA approach using generic TLA primers for haplotyping multiple samples without prior knowledge of SNV positions does not fit the requirements for reliable genotyping and phasing of pharmacogenes. Furthermore, the longer reads generated by Nanopore sequencing could not resolve this issue. First, the TLA workflow is costly when executed. Second, the protocol is time-consuming, as it takes four days for the TLA-PCR amplicons to be generated. Furthermore, the reads are not equally distributed on the target when using the TLA approach. Therefore, sequencing at a higher depth is needed compared with other approaches. However, even when more than 1000X average depth would be reached, some target positions would still not be covered, and some regions would be missed due to short fragments that could not be mapped unambiguously in this setup. This additionally emphasizes the importance of selecting the correct viewpoints when executing this TLA approach, which should ideally be optimized per patient or be located near high-frequency SNVs in the population ^12^. Alternatively, the PCR enrichment step could be replaced by a hybrid capture panel allowing more viewpoint positions to be included while generating more even coverage over the gene of interest. Considering this, using the method as described for screening many patient samples at once is seriously complicated. Altogether, although the TLA technology has already proved its usefulness in other contexts, its inherent drawbacks make this technology unsuitable for reliable genotyping and phasing of pharmacogenes such as those studied in this research.

## Conclusion

This study sequenced TLA libraries of four different pharmacogenes, *CYP2D6*, *CYP2C19*, *CYP1A2*, and *BRCA1*, using Illumina and Nanopore sequencing technologies. Only 24 and 38% of the sequenced nucleotides mapped on target for Illumina and Nanopore sequencing, respectively. *CYP2D6* could be covered entirely when four viewpoints were used. Except for *CYP1A2,* the genes were covered for more than 95% with the viewpoints in this study. The *CYP2D6* Nanopore dataset of viewpoint 4 resulted in the correct phased genotype compared to Rubben et al.^25^. In the other datasets, only a minor part of the variants was called and phased correctly compared to reference sets. Overall, variant calling was more accurate in the Illumina datasets, whereas more accurate phasing was obtained with the Nanopore dataset. Only for *CYP2D6*, a gene with homologous pseudogenes causing difficulties in mapping Illumina sequencing reads, the long Nanopore reads improved the variant calling compared with Illumina sequencing. These results could be improved if more viewpoints near heterozygous variants were used. However, as applying these optimizations would translate into a higher cost and more time-consuming protocol, the TLA workflow is considered unsuitable as a generalized assay for reliable genotyping and phasing of important pharmacogenes such as the ones in this study.

## Supplementary Information

Supplementary Information.

## Data Availability

The datasets generated and analyzed during the current study are available as BioProject, PRJNA853301. The used code is available on GitHub: https://github.com/laurentijntilleman/TLA_haplotyping_illumina_nanopore.
