# Supplementary material for: Haplotyping pharmacogenes using TLA combined with Illumina or Nanopore sequencing
Source: Sci Rep. 2022 Oct 22;12:17734. doi: 10.1038/s41598-022-22499-0 (PMC9587992; doi:10.1038/s41598-022-22499-0)
Supplement: Supplementary file 1 — Supplementary Information. [file 41598_2022_22499_MOESM1_ESM.docx]

Supplemental material:

Haplotyping pharmacogenes using TLA combined with Illumina or Nanopore sequencing

Laurentijn Tilleman*^,1^, Kaat Rubben*^,1^, Wim Van Criekinge^2^, Dieter Deforce^1^, Filip Van Nieuwerburgh^1,†^

*: equal contribution

^1^: Laboratory of Pharmaceutical Biotechnology, Ghent University,
Ottergemsesteenweg 460, 9000 Ghent, Belgium

^2^: Laboratory of Bioinformatics and Computational Genomics, Ghent University,
Coupure Links 653, 9000 Ghent, Belgium

^†^: Correspondence: filip.vannieuwerburgh@ugent.be

Table S1 TLA-primers on the different viewpoints for the different genes. * denote phosphorothioate bonds.

| Viewpoint primers | Sequence |
| --- | --- |
| BRCA1_F | CATTACTGTAGAAGTTCCCTA*A*A |
| BRA1_R | ACCATTGCTGTTCCTTCT*A*A |
| CYP1A2_F | TTTGACTCATCCAGCT*G*G |
| CYP1A2_R | CTTTGAGCAAGGGTAGGA*T*T |
| CYP2D6_1_F | GGATGTCATATGGGTCAC*A*C |
| CYP2D6_1_R | TTCCGCATCCCTAAGGT*A*G |
| CYP2D6_2_F | GGGACTTTGTACTCCATAA*C*A |
| CYP2D6_2_R | CAGTGACCCGGTTCAA*A*C |
| CYP2D6_3_F | TTTCCCAGATGGGCTC*A*C |
| CYP2D6_3_R | GAGGGAGGAAGGGTAC*A*G |
| CYP2D6_4_F | CACCCACACTGAGCTT*A*C |
| CYP2D6_4_R | AAGGTGGATGCACAAAG*A*G |
| CYP2C19_1_F | GTAAAGGGCTGCGATTAG*T*A |
| CYP2C19_1_R | GAAGTTCAATTTTCACGG*C*T |
| CYP2C19_2_F | AGACAGTAGAAGGATGGG*T*A |
| CYP2C19_2_R | TCTCAGACCTAGAAGAC*C*C |
| CYP2C19_3_F | GCCCATCTTGTCATTGTA*A*A |
| CYP2C19_3_R | TGATGTGATGCAGAATTG*A*C |
| CYP2C19_4_F | CAGGAATGTTGTGATTTTGT*T*G |
| CYP2C19_4_R | CCAGTGATGGTAGAGGG*T*A |


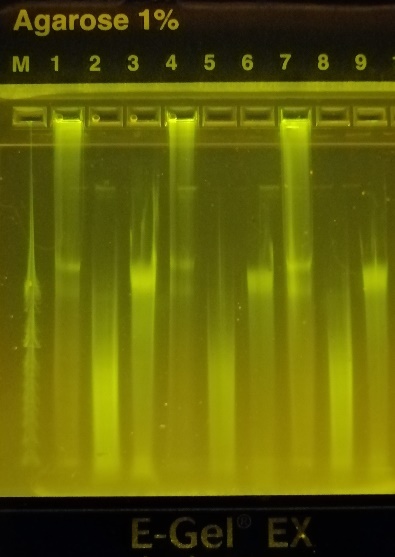


Figure S1 Length measurement of the undigested, digested and ligated DNA during the TLA protocol. M: molecular marker; lines 1, 4, and 7: undigested DNA; lines 2, 5, and 8: DNA after digestion with NlaII; lines 3, 6, and 9: ligated DNA.


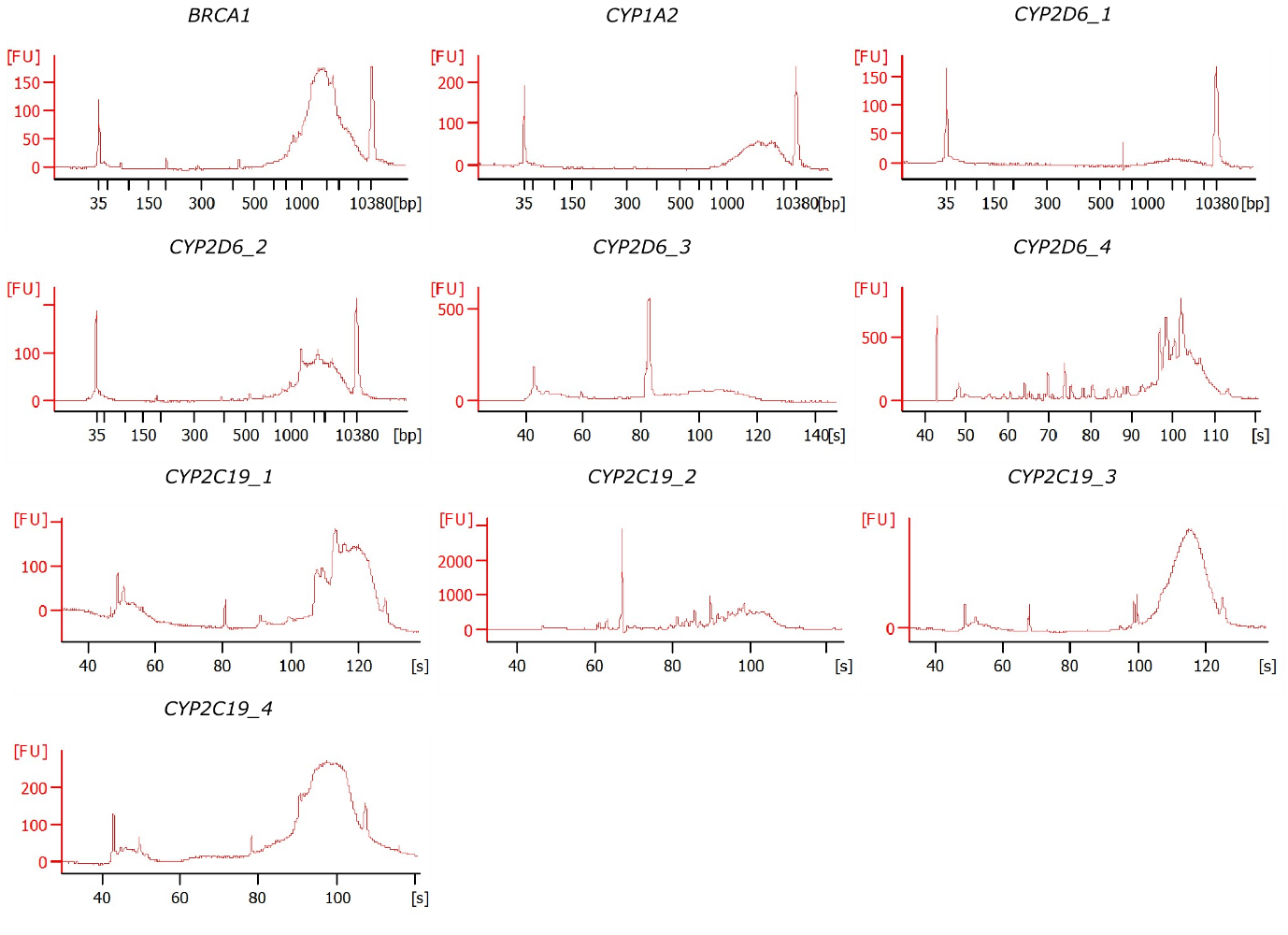


Figure S2 Bioanalyser traces of the TLA-PCR products from the different viewpoints. The x-axis represents the running time. For BRCA1, CYP1A2, CYP2D6_1, and CYP2D6_2, this running time is translated into fragment lengths. The y-axis represents the relative fluorescent signal.

Table S2 Overview of the sequencing pools. Illumina pools were sequenced on Illumina MiSeq Micro run PE 2x151; Nanopore pools 1 and 2 were sequenced on a Flongle flow cell; Nanopore pool 3 was sequenced on a MinION flow cell.

| Illumina pool 1 (equimolar) | *BRCA1, CYP1A2, CYP2D6_1, CYP2D6_2* |
| --- | --- |
| Illumina pool 2 (equimolar) | *CYP2D6_3, CYP2D6_4, CYP2C19_1, CYP2C19_2, CYP2C19_3, CYP2C19_4* |
| Nanopore pool 1 (equimolar) | *BRCA1, CYP1A2, CYP2D6_1, CYP2D6_2* |
| Nanopore pool 2 (equimolar) | *CYP2D6_3, CYP2D6_4, CYP2C19_1, CYP2C10_2, CYP2C19_3, CYP2C19_4* |
| Nanopore pool 3 (equimolar) | *BRCA1, CYP1A2, CYP2D6_1, CYP2D6_2, CYP2D6_3, CYP2D6_4, CYP2C19_1, CYP2C10_2, CYP2C19_3, CYP2C19_4* |


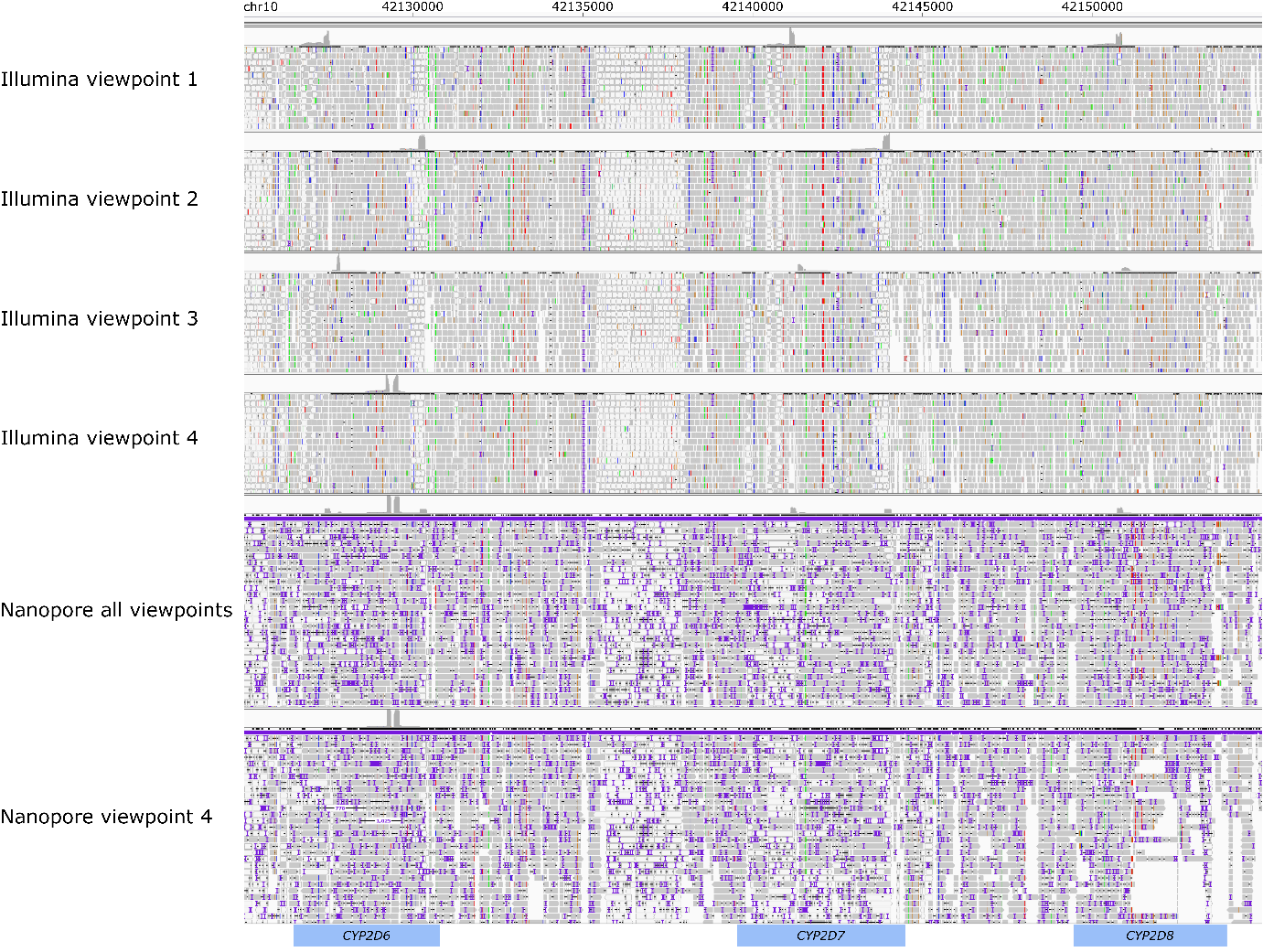


Figure S3 IGV images of the Illumina and Nanopore sequencing reads of the different viewpoints mapped on the CYP2D6 locus. For each dataset, the graph is split into two parts. The upper part represents the sequencing depth. A high sequencing depth is observed in the regions where the reads from the viewpoints map. The lower part represents the individual reads. Grey bars represent unambiguously mapped reads; white bars represent ambiguously mapped reads; colored positions in the bars represent alternative nucleotides, insertions, and deletions in the read.


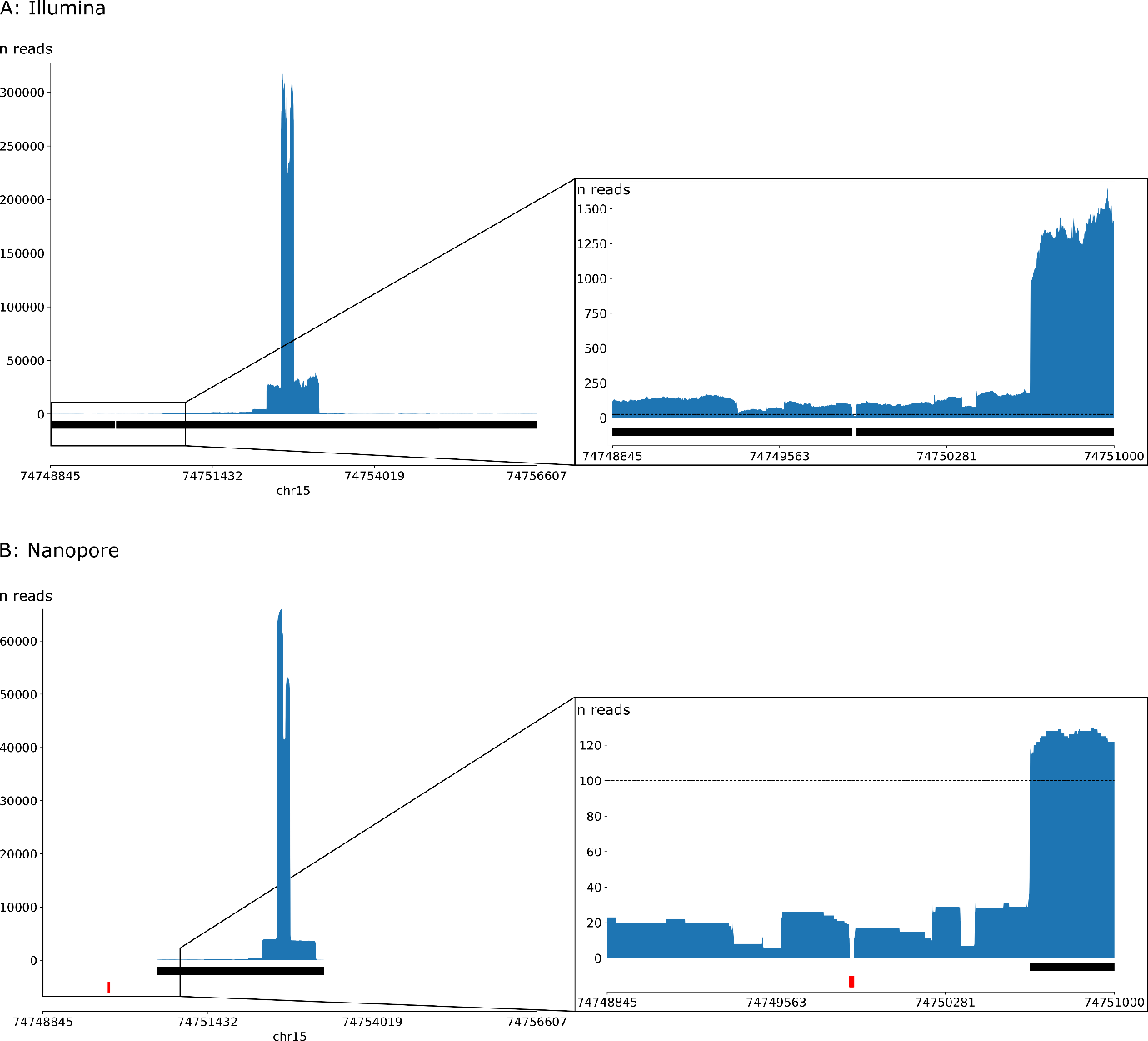


Figure S4 Coverage plots for CYP1A2. A: Illumina sequencing data; B: Nanopore sequencing data. Blue surfaces represent the depth on each position in CYP1A2; dark bars under the graphs define the regions with a minimal depth, 25 reads for Illumina sequencing and 100 reads for Nanopore sequencing; red bars under the graphs represent regions with no reads.


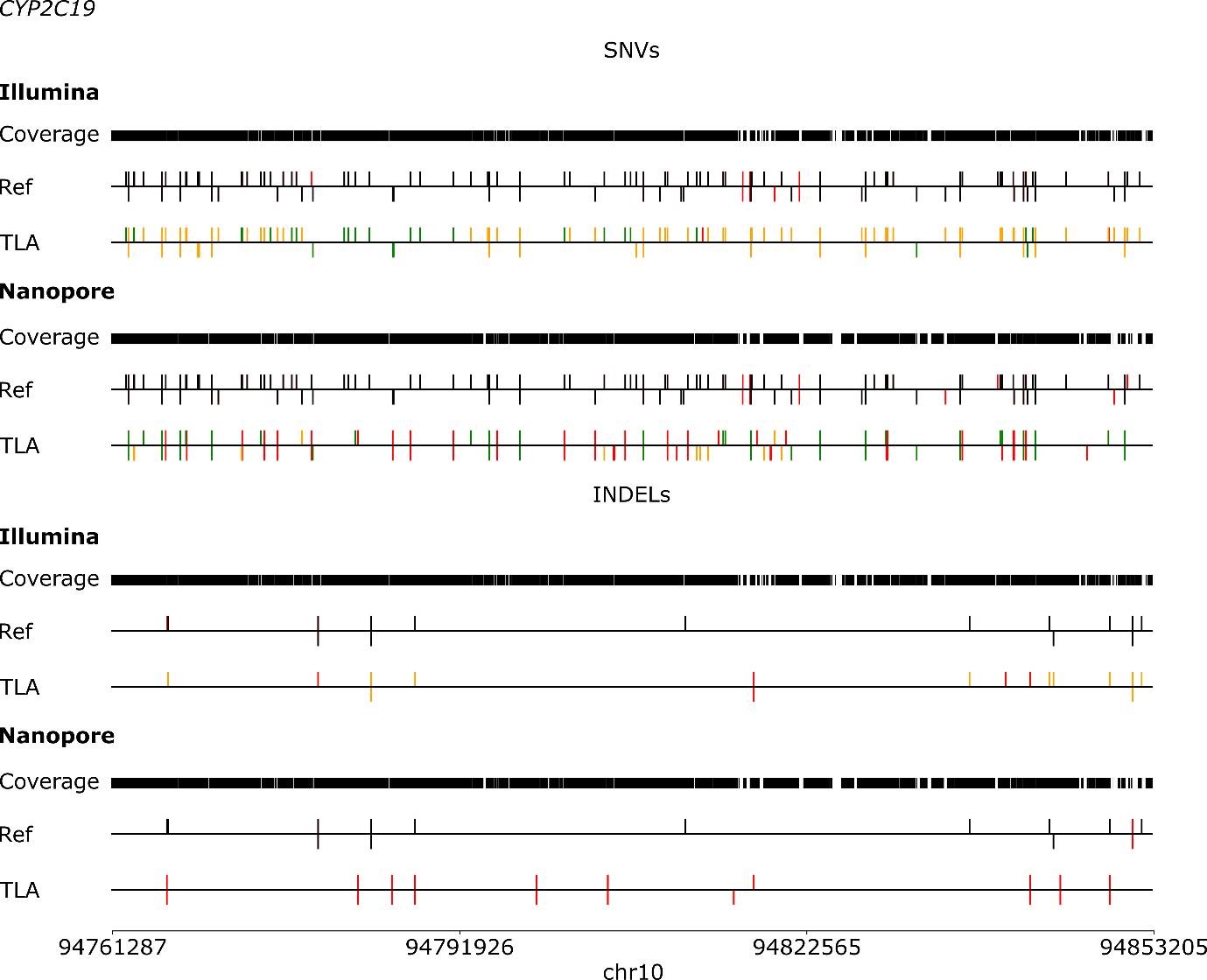


Figure S5 Coverage, variant calling, and phasing results for CYP2C19. Coverage: bars indicate where sufficient depth is reached: 25X for Illumina and 100X for Nanopore. Ref: The reference line shows the variants present in the reference set of Krushe et al. [23]. Red-colored variants in the reference correspond to variants wherefore not sufficient depth is reached in the sample. Bars plotted at the same side of the reference line (top or bottom) represent variants from the same allele. TLA: red bars represent discordant variants, orange bars represent variants that were phased incorrectly or that could not be phased, and green bars represent concordantly called and phased variants.


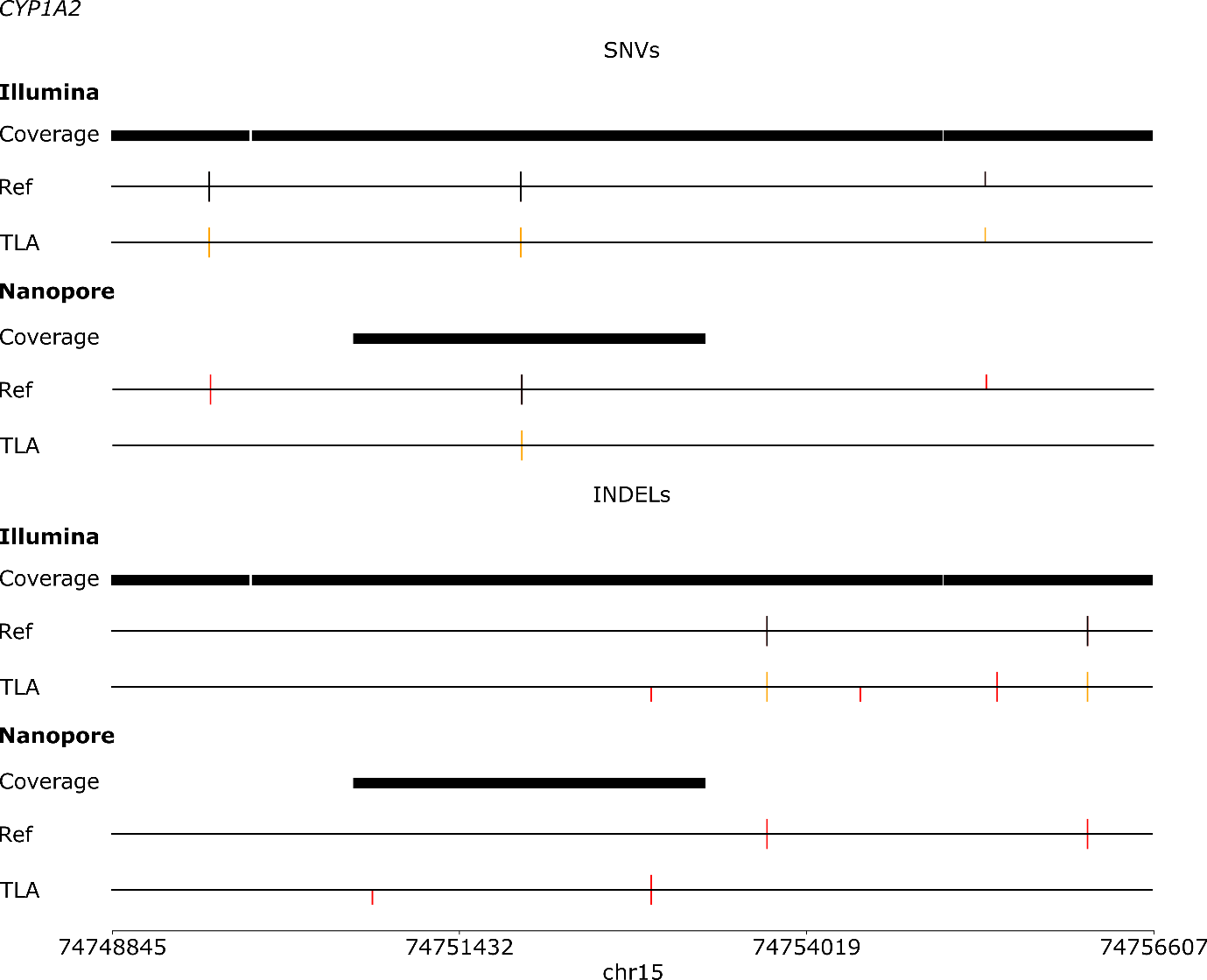


Figure S6 Coverage, variant calling, and phasing results for CYP1A2. Coverage: bars indicate where sufficient depth is reached: 25X for Illumina and 100X for Nanopore. Ref: The reference line shows the variants present in the reference set of Krushe et al. [23]. Red-colored variants in the reference correspond to variants wherefore not sufficient depth is reached in the sample. Bars plotted at the same side of the reference line (top or bottom) represent variants from the same allele. TLA: red bars represent discordant variants, orange bars represent variants that were phased incorrectly or that could not be phased, and green bars represent concordantly called and phased variants.


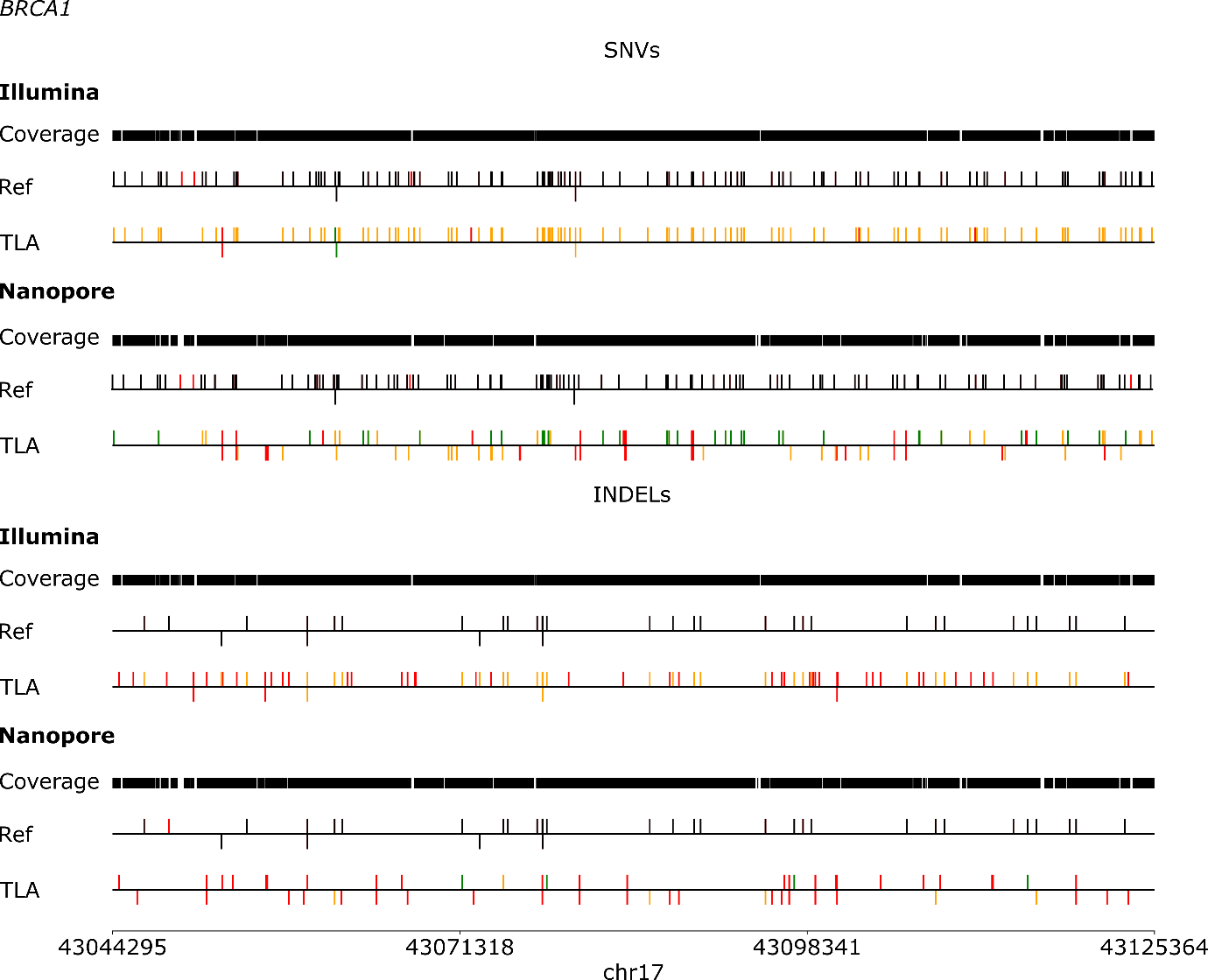


Figure S7 Coverage, variant calling, and phasing results for BRCA1. Coverage: bars indicate where sufficient depth is reached: 25X for Illumina and 100X for Nanopore. Ref: The reference line shows the variants present in the reference set of Krushe et al. [23]. Red-colored variants in the reference correspond to variants wherefore not sufficient depth is reached in the sample. Bars plotted at the same side of the reference line (top or bottom) represent variants from the same allele. TLA: red bars represent discordant variants, orange bars represent variants that were phased incorrectly or that could not be phased, and green bars represent concordantly called and phased variants.
